# Prone positioning in the elderly extends perioperative process times: a retrospective analysis

**DOI:** 10.3205/iprs000126

**Published:** 2018-12-06

**Authors:** Joerg Schnoor, Christoph E. Heyde, Mary Niese-Anke, Steffen Friese, Thilo Busch, Jan-S. Jarvers

**Affiliations:** 1 Department of Anaesthesia and Intensive Care Medicine, Collm Klinik Oschatz, Germany; 2 Department of Anaesthesia and Intensive Care Medicine, University Hospital Leipzig, Germany; 3 Department of Orthopaedics, Trauma Surgery and Reconstructive Surgery, University Hospital Leipzig, Germany

**Keywords:** elderly, demographic change, cervical fracture, process times, patient positioning

## Abstract

**Objective:** Cervical bone fractures describe a predominant trauma in the elderly. With demographic change, prone patient positions might create further stress on personnel resources. Therefore, the aim of this study was to conduct an age-related analysis of pre- and intraoperative process times in patients with cervical fractures.

**Methods:** We reviewed all schedules with cervical spine surgery performed at a tertiary hospital. Two different operative patient positions were specified: prone and supine. We retrospectively analysed three study groups: comparison group (group 1: ≤59 years of age), old patients (group 2: 60–79 years), and very old patients (group 3: ≥80 years). We recorded date and kind of surgery, biometric data, and process times by screening recordings of internal software programs (COPRA^®^ and SAP 710^®^). Group comparisons were conducted using the Kruskal-Wallis test with Dunn’s post hoc test and Bonferroni correction, Pearson’s chi-square test, and the Mann-Whitney U test, as required.

**Results:** 330 patients (202 male; 128 female) were analysed. The number of patients in the resulting age-dependent groups 1–3 were n=102, n=123, and n=105, respectively. Patients of increasing age and in supine position showed a continuous increase in the time needed for anaesthesia induction (mean between 4 and 8 minutes (p<0.05). When compared to patients in supine position, this time further increased on average by 6 minutes (p<0.05) in old but prone patients. In old and very old patients, getting a patient into a prone position was associated with a time demand between 10 and 12 minutes (p<0.01), respectively. While time for surgery age-dependently decreased in patients that were supine positioned (p<0.001), surgery time was prolonged between 34 and 104 minutes (p<0.05) in patients that were prone.

**Conclusion:** With prone position both anaesthesia-controlled and surgical-controlled times extended in patients of increasing age. With regard to demographic change, this aspect should be considered for future revenue calculations in flat-rate remuneration systems.

## Zusammenfassung

**Ziel:** Frakturen der Halswirbelsäule sind ein häufiges Trauma bei älteren Menschen. Angesichts des demografischen Wandels könnte eine Zunahme an Bauchlagerungen dieser Patienten zu einer weiteren Belastung der Personalressourcen führen. Ziel dieser Studie war es daher, eine altersbezogene Analyse der perioperativen Prozesszeiten bei Patienten mit Frakturen der Halswirbelsäule durchzuführen.

**Methoden:** Die OP-Pläne aller Halswirbelsäulenoperationen einer Universitätsklinik wurden über einen Zeitraum von 10 Jahren retrospektiv überprüft. Zwei unterschiedliche Patientenlagerungen wurden spezifiziert: Bauch- und Rückenlage. Insgesamt wurden drei altersabhängige Patientengruppen generiert: Vergleichsgruppe (Gruppe 1: ≤59 Jahre), ältere Patienten (Gruppe 2: 60–79 Jahre) und sehr alte Patienten (Gruppe 3: ≥80 Jahre). Erfasst wurden das Datum und die Art der Operation, biometrische Daten und die perioperativen Prozesszeiten anhand interner Softwareprogramme (COPRA^®^ und SAP 710^®^). Die Gruppenvergleiche erfolgten unter Verwendung des Kruskal-Wallis-Tests mit Dunns Post-hoc-Test und Bonferroni-Korrektur, Pearson's Chi-Quadrat-Test und dem Mann-Whitney-U-Test.

**Ergebnisse:** 330 Patienten (202 männlich, 128 weiblich) wurden analysiert. Die Patientenanzahl der altersabhängigen Gruppen (1–3) lag bei n=102, n=123, bzw. n=105. Patienten mit zunehmendem Alter und Rückenlage zeigten eine zeitliche Verlängerung der Anästhesieeinleitung (Mittelwert 4 Minuten bzw. 8 Minuten, p<0.05). Im Vergleich mit Patienten in Rückenlage nahm diese Prozesszeit bei älteren Patienten in Bauchlage im Durchschnitt um weitere 6 Minuten (p<0.05) zu. Die Bauchlagerung als solche dauerte zwischen 10 Minuten bei älteren Patienten (p<0.01) und 12 Minuten bei sehr alten Patienten. Im Vergleich zu gleichaltrigen Patienten in Rückenlage dauerten die operativen Maßnahmen in Bauchlage zwischen 34 und 104 Minuten (p<0.05) länger.

**Schlussfolgerung:** Durch die Bauchlagerung verlängerten sich sowohl die Anästhesie kontrollierten als auch die Chirurgie kontrollierten Prozesszeiten bei Patienten mit zunehmendem Alter. Im Hinblick auf den demografischen Wandel sollte dieser Aspekt für künftige Erlösberechnungen zeitnah berücksichtigt werden.

## Introduction

With a share of 25% of all lesions, cervical bone fractures are one of the most common injuries of the cervical spine. For example, odontoid fractures describe a predominant trauma in the elderly that is frequently promoted by osteoporosis and degenerate joint changes [1], [2], [3]. As a first therapeutic option, surgical stabilization is performed while the patient is either in prone or supine position. To bring an anaesthetized patient into a prone position can be a time consuming process that might be even more complex in patients of increasing age. 

While the operating room (OR) is a cost-intensive environment, effective scheduling of the surgical suite has already become an essential prerequisite for efficient OR management [4]. With demographic change, to shift an aged patient into a prone position possibly creates further stress on personnel resources. Therefore, this study aimed to conduct an age-related analysis of pre- and intraoperative process times in patients with cervical fractures.

## Methods

This retrospective study has been accepted by the local ethics committee (235-13-26082013). We reviewed all schedules with cervical spine surgery at the University Hospital Leipzig from 2002 to 2012. Two different operative patient positions were specified: prone (posterior stabilization) and supine (anterior stabilization). We recorded the patient’s age, date of surgery, biometric data, and process times by screening recordings of internal software programs (COPRA^®^ and SAP 710^®^). All data were transferred into electronic data (Microsoft, Excel^®^) for statistical analysis. 

### Surgical techniques and patient positioning

The kind of surgical treatment depends on the injury. Supine patient positioning was used for Anderson Type II odontoid fractures. Here, an anterior screw fixation of the dens (odontoid screw fixation [OSF]) was performed. If these injuries were associated with arthrosis of the C1/2-joint and/or a fracture of the atlas, a transarticular atlantoaxial fixation with odontoid fusion (TAFOF) was made. Injuries with sub C2 diagnosis associated with disc injuries were mainly treated via anterior cervical decompression and fusion (ACDF). 

A prone patient position became necessary in cases of atypical odontoid fractures, dens dislocations >2 mm, or a pseudarthrosis of the dens, in order to facilitate a posterior stabilization of the C1/2-joint. In addition, all injuries with neurological deficits required a posterior stabilization and decompression in a prone position. 

### Age-dependent groups

We divided all patients by their age at the time of surgery into three groups: comparison group (group 1: ≤59 years), old patients (group 2: 60–79 years), and very old patients (group 3: ≥80 years). 

### Definition of process times

The process times correspond to those defined in the glossary of perioperative process times of the German Society of Anaesthesiology and Intensive Care Medicine (Table 1 (Tab. 1), [5]).

### Statistical analysis 

Statistical analysis was computer based, using SPSS^®^ Statistics, Version 20 (IBM Corp., Armonk, NY). Comparisons between age groups were conducted using the Kruskal-Wallis test with Dunn’s post hoc test and Bonferroni correction and Pearson’s chi-square test. The Mann-Whitney U test was used for a comparison between prone and supine patient position. Correlation analysis was performed using the Spearman’s rank correlation coefficient. We displayed data as an arithmetic mean and standard deviation. Statistical significance was accepted at two-sided p-values <0.05.

## Results

**Figure 1:**
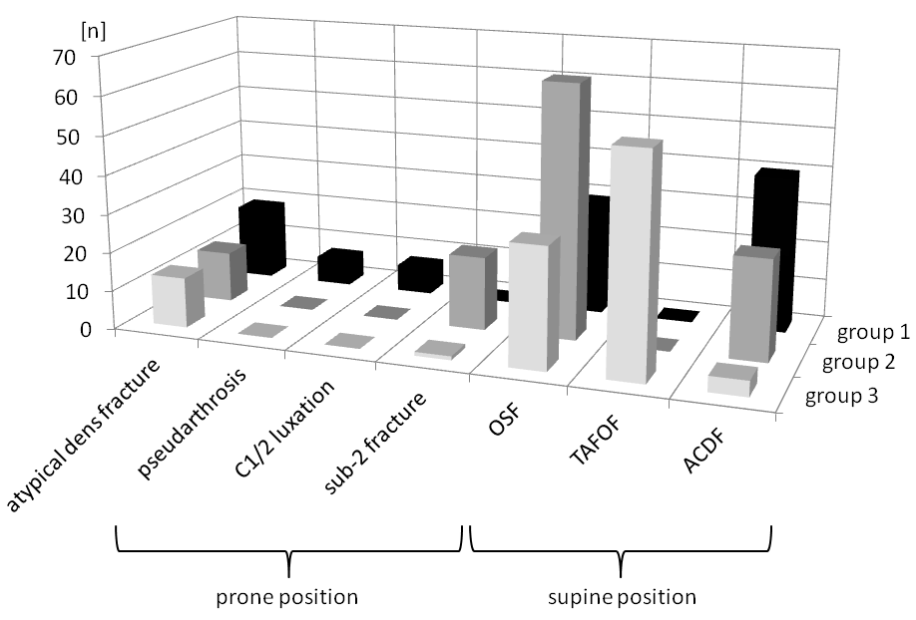
Number of age related surgical treatments and patients’ positioning ACDF: anterior cervical decompression and fusion; OSF: anterior odontoid screw fixation; TAFOF: anterior transarticular atlantoaxial fixation and odontoid fusion

In total, 330 patients (202 male; 128 female) were analysed. The number of patients in the resulting age-dependent groups 1–3 were n=102, n=123, and n=105, respectively. Of the patients, 24% were in prone position (n=79). Many more cervical spine operations were performed in supine position (n=251, p=0.005), and the number increased with increase in age (controls vs. very old patients, p=0.001). Controls and old patients comprised mostly male patients (p<0.001) that had suffered an accident in the household (52%) or due to road traffic (28%). In contrast, very old patients were mostly female (p<0.001) and threatened by a low-velocity fall (63%). Table 2 (Tab. 2) presents the distribution of patients’ age, gender, and positioning in the respective groups. The shares of the different surgical procedures are shown in Figure 1 (Fig. 1). 

### Age-dependent process times independent of patient positioning 

Values of respective process times for each age-dependent group are presented in Table 3 (Tab. 3). Statistically significant differences between the groups were found for both delta 2 (time for anaesthesia induction) and delta 5 (time for surgery).

The time needed for anaesthesia induction (delta 2) increased with increase in age. Between group 1 and old patients, induction time increased on average by 5 minutes (p=0.029). Between group 1 and very old patients (group 3), this time increased on average by 7 minutes (p<0.01, Table 3 (Tab. 3)). The age-dependent extension of the induction time was associated with a higher share of patients monitored by arterial cannulation (p<0.001) and central venous catheter (p<0.001). The rate of awake tracheal intubation was comparable between the groups (p=0.064). 

In total, the time for surgery (delta 5) shortened with increase in age. Time for surgery decreased between group 1 and old patients (group 2) on average by 22 minutes (p=0.019). Compared with group 1, this time decreased on average by 49 minutes (p<0.001) in very old patients (group 3, Table 3 (Tab. 3)). The age-dependent shortage of the time for surgery was associated with a higher rate of odontoid fractures (Anderson Type II, group 1: 36.3%; group 2: 63.4%; group 3: 95.2%; p<0.01, Figure 1 (Fig. 1)), which were mostly treated by anterior screw fixation of the dens (OSF) or TAFOF with patients being supine positioned. 

A deduced time-share out of the anaesthesia induction time and the operation time increased with age. In groups 1–3, this time-share was 16%, 24%, and 34%, respectively. Another time-share also increased in an age-dependent manner: The share of preoperative surgical measures (delta 4) in relation to the operating time was 35%, 42%, and 49%, respectively. 

### Age-dependent process times in patients in supine versus prone position

**Figure 2:**
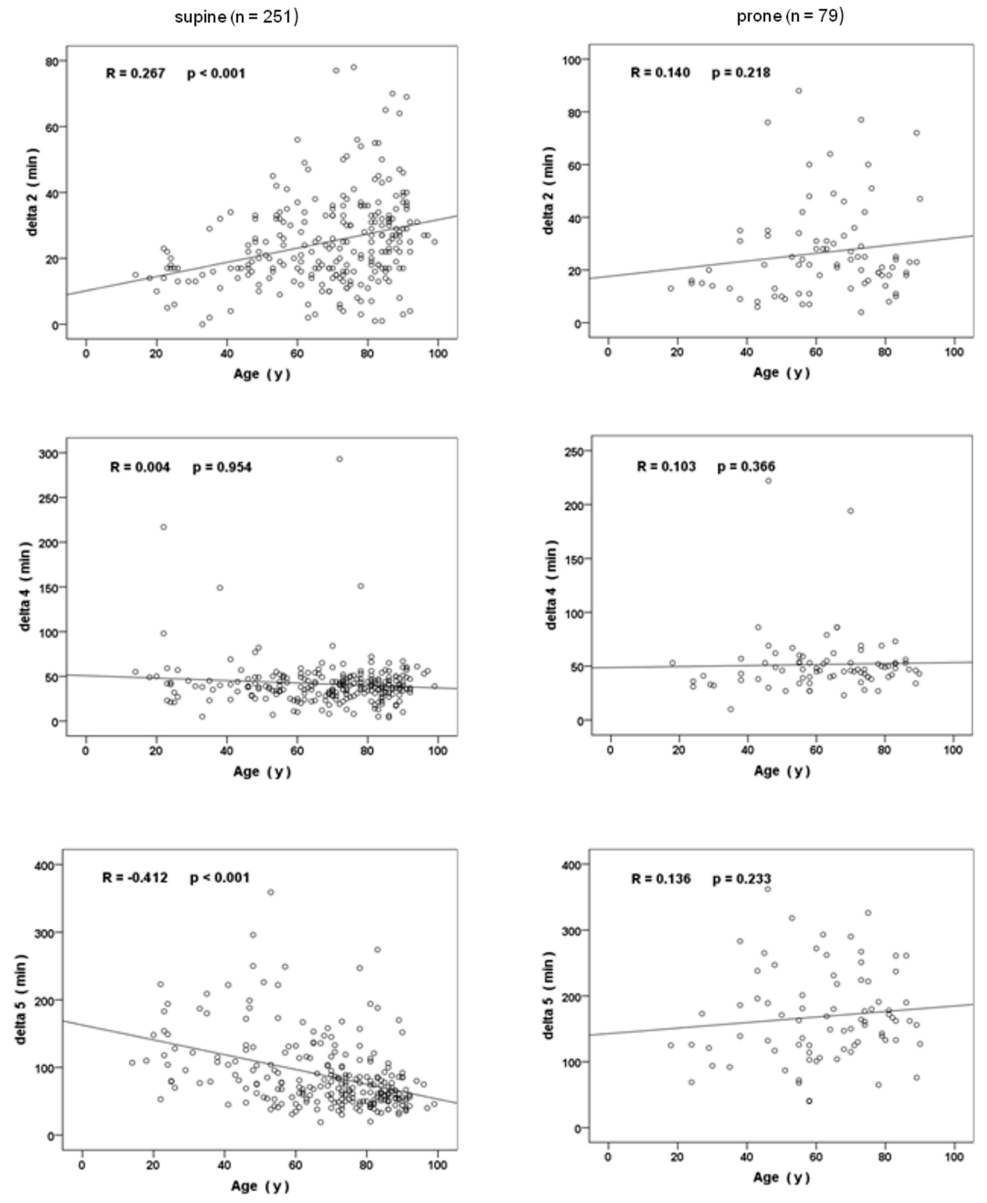
Age related process times in patients (supine versus prone patient positioning) given as regression line R = Spearman coefficient of determination and p = statistical significance of correlation

Concerning both patients’ age and positioning (supine versus prone), the resulting process times are pictured in Figure 2 (Fig. 2). Anaesthesia induction time increased in old patients that were prone, while surgical time was significantly prolonged in old as well as very old patients: Comparable to the aforementioned data, patients of increasing age and in supine position showed a continuous increase in the time needed for anaesthesia induction (delta 2) on average between 4 minutes (group 2 versus group 1, p<0.001) and 8 minutes (group 3 versus group 1, p<0.05), respectively, (Table 4 (Tab. 4)). When compared to supine patients of the same age, delta 2 further increased by 6 minutes (p<0.05) in old patients that were prone, while delta 2 shortened in prone and very old patients (group 3, p<0.05). 

Getting a patient into a prone position was associated with a prolonged process time that lasted from anaesthetists’ release of the patient for surgery until the start of surgery (delta 4). In prone patients, this process was prolonged and lasted on average 12 minutes more (p<0.01) in old and 10 minutes (p<0.01) more in very old patients, respectively (Table 4 (Tab. 4)).

With increasing age, time for surgery (delta 5) continuously decreased in patients that were treated in a supine position (p<0.001). In contrast, patients that were prone showed an increase of delta 5 in an age-dependent manner (p<0.001). When compared to supine position and group-by-group, prone patients showed a prolonged surgical time between 34 and 104 minutes on average (p<0.05, Table 4 (Tab. 4)).

## Discussion

This retrospective analysis shows that cervical fractures primarily affect male patients until the age of 80. It was not until the ninth decade of life that women were predominant. While younger patients mostly suffered accidents in the garden or due to road traffic, octogenarians mostly demonstrated odontoid fractures due to low-velocity falls. Elderly patients were associated with a prolongation of the anaesthesia induction time that seemed to be due to an extended use of cardiovascular monitoring. Getting an aged patient into a prone position took about 10 minutes. 

Surgical theatres are cost-intensive environments, which should be managed efficiently by brief scheduling promoting short case durations with minimized non-operative times [4]. In short, surgeons’ experiences are main factors in lessening the former, while the latter mostly results from straightforward measures during anaesthesia-controlled procedures [6]. Normally, the time-share of anaesthesia induction in relation to the operating time seems to be about 10% [4]. We found an age-dependent increase of this ratio up to 34% in very old patients. Another time-share of preoperative surgical measures in relation to the operating time increased up to 49% in the elderly. In the future, more patients with increasing age will need prolonged time slots to get anesthetized, positioned, and prepared for surgery.

As part of the anaesthesia-controlled time, anaesthesia induction time was prolonged in patients of increasing age. Frequently, aged and morbid patients demonstrate a higher class in the American Society of Anaesthesiologists (ASA) physical status. An associated ASA class IV triples the anaesthesia preparation time compared with ASA physical status class I [6], [7]. This is partly due to more time-consuming preparation. For example, comorbidities more often require invasive hemodynamic monitoring. Our results show that additional monitoring (e.g. arterial catheter and central venous catheter) in old and very old patients required a few minutes. Silber et al. found that an increased body mass index, paraplegia, hypertension, diabetes or coagulation disorders were other typical reasons to prolong procedure times [8]. When compared to asleep tracheal intubation techniques, awake intubation added approximately eight more minutes [9]. Contrarily, we did not find any indication that intensified airway management had a significant effect on process time. This might be because most patients with cervical fractures were fibre-optically intubated, independent of patients’ age. In this regard, another time-share might be required for tertiary hospitals that are bound to staff training. Both teaching a resident and supervising several operating rooms by one consultant might require further time during anaesthesia induction [10]. 

We hypothesized that shifting an anesthetized patient into a prone position is a time-consuming process. Indeed, getting a patient into a prone position required about ten minutes. In those cases, time for surgery additionally increased, with total case time markedly prolonged. On the other hand, our results show that the older the patients were, the more often they were operated on in a supine position. Thus, octogenarians mostly suffered odontoid fractures, which were treated by an anterior screw fixation or transarticular atlantoaxial fixation. Both techniques, performed in supine patient position, were associated with an increase in anaesthesia-controlled time, but the time for surgery markedly decreased. In consequence, an extension of an anaesthesia-controlled time was more than compensated for by a shortened time for surgery. 

In conclusion, when older patients with a fractured cervical spine were treated in a prone position, both anaesthesia-controlled and surgical-controlled times were markedly prolonged. With regard to demographic change, this aspect should be considered for future revenue calculations in flat-rate remuneration systems.

## Notes

### Competing interests

The authors declare that they have no competing interests.

### Ethics approval

This study was approved by the local ethics committee (235-13-26082013).

## Figures and Tables

**Table 1:**
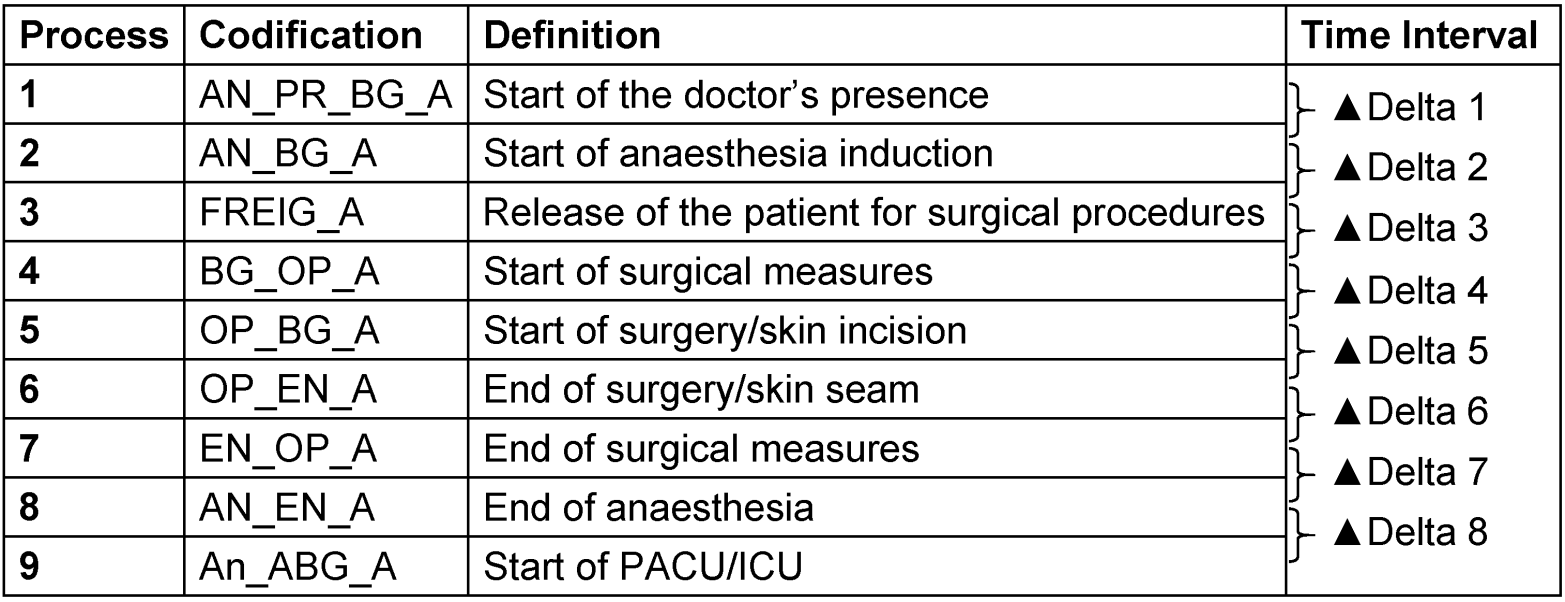
Classification of perioperative processes according to the recommendations of the German Society of Anaesthesiology and Intensive Care Medicine [5] and the resulting time intervals PACU = Post-Anaesthesia Care Unit; ICU = Intensive Care Unit

**Table 2:**
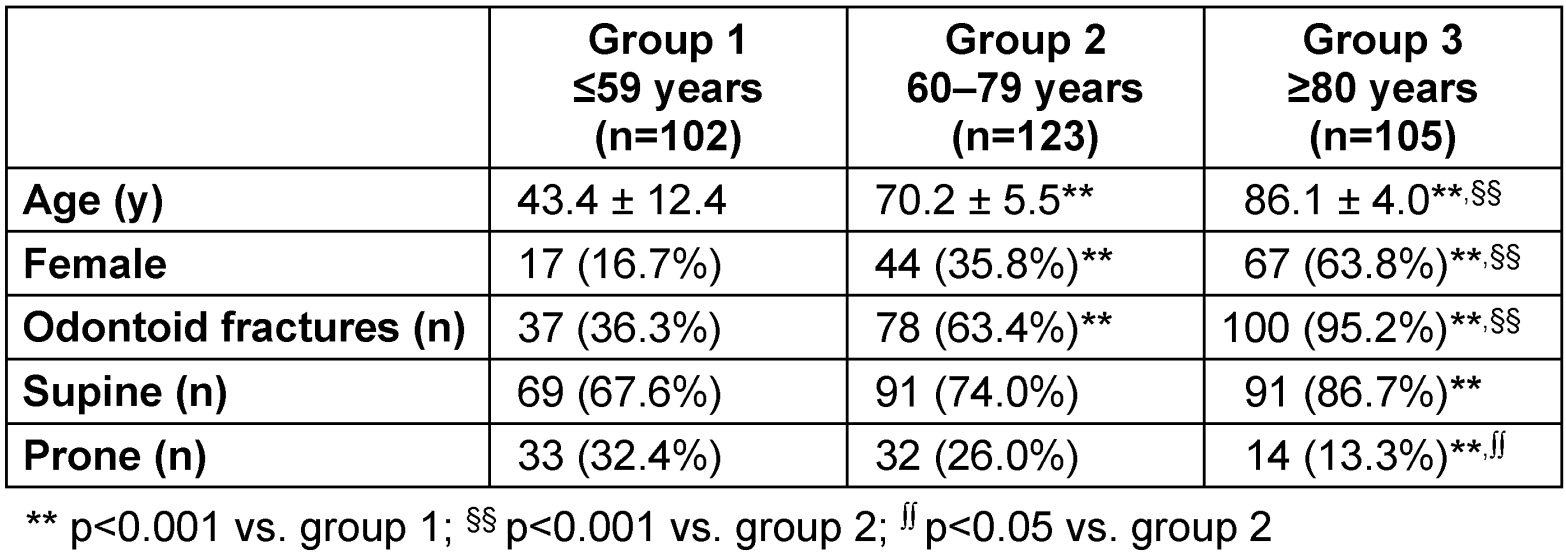
Distribution of patients’ age, gender, operative positioning, and surgical procedure Data are given as mean ± standard deviation. According to the Pearson’s chi-squared test, groups 1–3 were analysed for statistically significant differences. Supine = supine position; prone = prone position. P-values refer to Kruskal-Wallis test, significance symbols are based on Dunn’s post hoc test with Bonferroni correction.

**Table 3:**
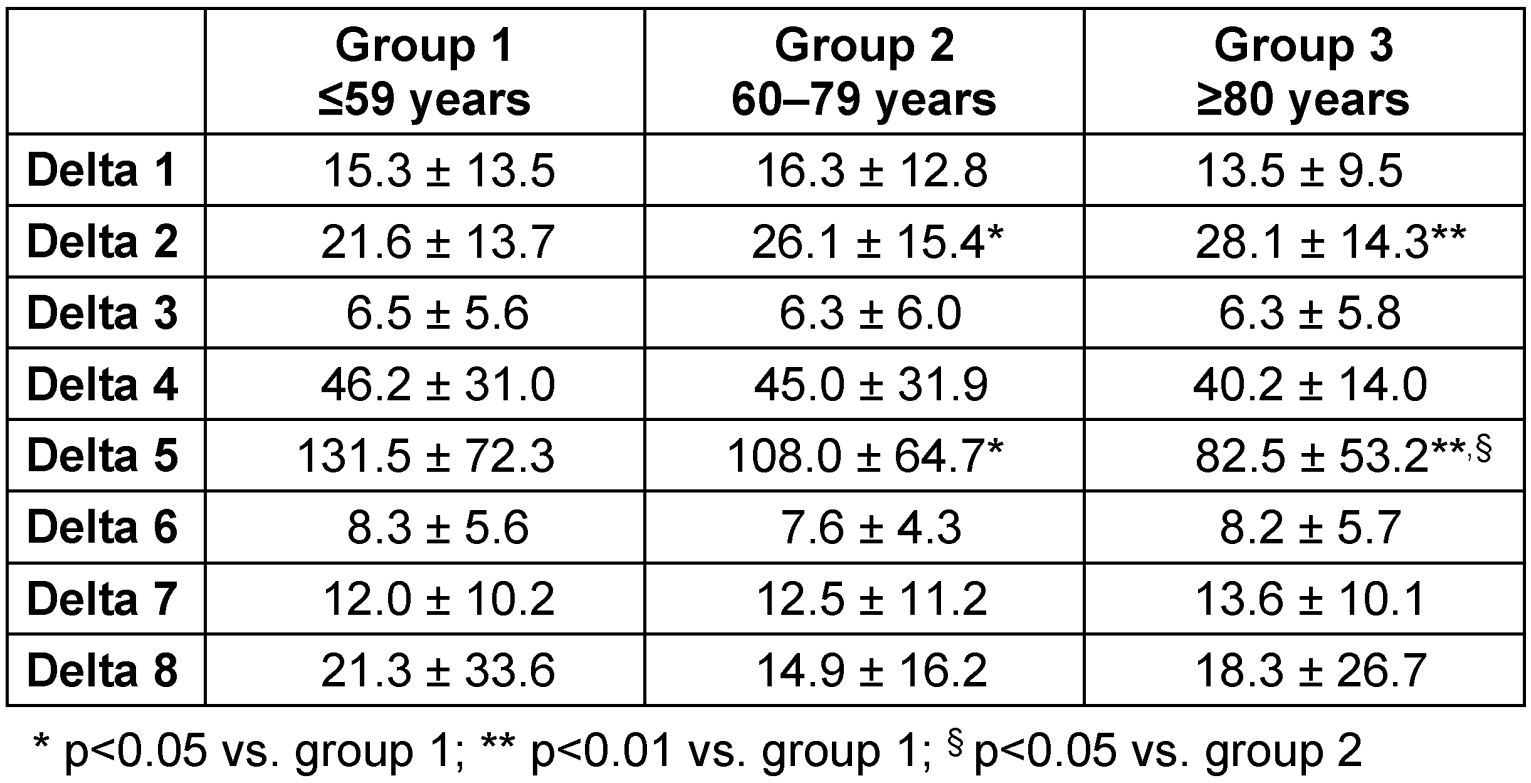
Pre- and intraoperative process times in age-dependent groups Data are given in mean ± standard deviation. P-values refer to the Kruskal-Wallis test, significance symbols are based on Dunn’s post hoc test with Bonferroni correction.

**Table 4:**
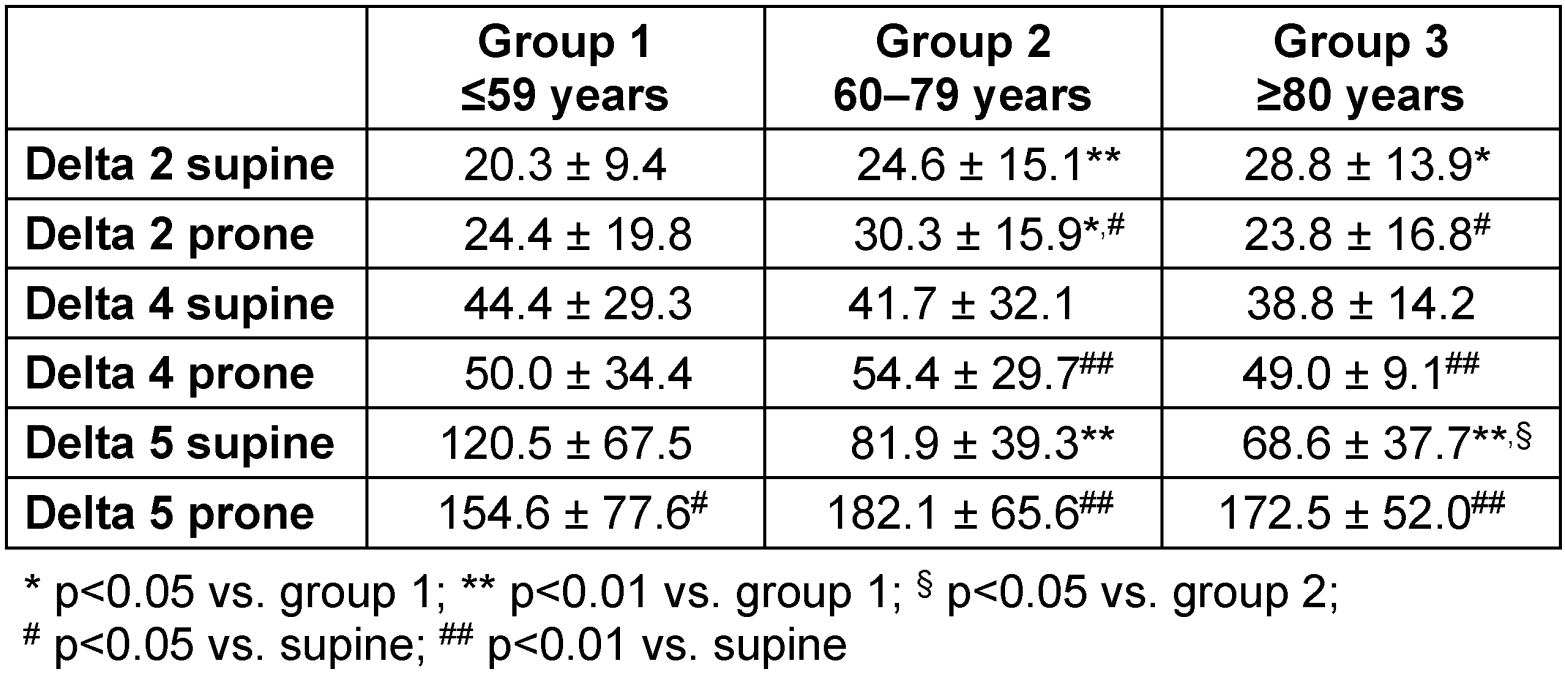
Pre- and intraoperative process times delta 2, delta 4, and delta 5 (minutes) of the age-dependent groups in patients with supine or prone position Data are given in mean ± standard deviation. According to the Mann-Whitney U test, patient positioning was analysed for statistically significant differences.
